# A concise pathophysiological model of acute appendicitis against the background of the COVID-19 pandemic

**DOI:** 10.3389/fped.2022.908524

**Published:** 2022-10-13

**Authors:** Marc Reismann

**Affiliations:** Department of Pediatric Surgery, Charité - Universitätsmedizin Berlin, Corporate Member of Freie Universität Berlin and Humboldt-Universität zu Berlin, Berlin, Germany

**Keywords:** acute appendicitis, children, COVID-19, pathophysiology, clinical implications

## Abstract

One of the most common clinical pictures has become the focus of attention during the COVID-19 pandemic: acute appendicitis with the associated diagnostics and therapy. The aim of the work is to show inconsistencies with regard to epidemiology, pathophysiology and therapy against the background of the pandemic with special attention to the conditions for children and to explain the pathophysiological processes that are likely to underlie the disease based on scientifically plausible models.

## Introduction

The Covid-19 pandemic has often been referred to metaphorically as a magnifying glass for various social, political and medical problems and peculiarities. It is not surprising that one of the most common clinical pictures has also become the focus of attention during the pandemic. However, the history of appendicitis is primarily a history of perception. Compared to pre-pandemic times, three fundamental aspects have come to the fore: pathophysiology, epidemiology and clinical aspects (diagnostics and therapy). With regard to all these aspects, various connections with pandemic conditions have been seen or at least claimed. In the current situation it is interesting that these three aspects are reflected in a condensed manner, allowing for a summary discussion of the same.

## The epidemiology of acute appendicitis under pandemic conditions

One of the first and most profound observations under pandemic conditions regarding acute appendicitis was a substantial change of the frequencies of affected patients. In one of the first works published on the subject in the title it was asked: Where did the patients go (1)? This was followed by the question in another publication: Where did all the appendicitis go (2)? The central observation in these published studies was that of a general decrease of the incidence of acute appendicitis during the pandemic compared with pre-pandemic times. This observation was repeatedly made in other studies (3–6). However, the central message in other studies was different: there it was particularly found that the rates of clinically complicated forms of the disease had *increased* during the pandemic. These results were primarily attributed to a delay in diagnostics and treatment of the patients, affecting adults and children (7–11). Thus, there is a clear difference between the statements of these studies. Even if all this work seems to provide new insights against the background of the pandemic, it reflects an old discussion that has not yet found a conclusion.

The central observation is that the *total* number of patients with acute appendicitis *decreased* irrespective of age group (1–6). As the number of patients with appendicitis in the particular years varies over the years – especially in bigger cities with more than one hospital -, information on total numbers is not easy to get: it is either necessary to collect data within a multicenter approach (1, 2, 5, 6) or to use huge central data bancs e.g., from health insurances (4). The second observation regarding the perception of an increase of patients with complicated appendicitis is based on proportions and not on total numbers. First, it has to be mentioned that particularly definitions are a crucial point here. In most of the studies on appendicitis definitions of complicated appendicitis seem to be similar, but in fact differ. Criteria in the particular studies included CT findings of phlegmon or abscess, intraoperative findings of perforation or abscess (1), rates of open appendectomy, percutaneous drainage, shock, mortality, hospital length of stay (2) or gangrene, perforation or peri-appendicular abscess (6), and others referred to the general surgical Clavien-Dindo classification (5). The most widely used definition of complicated appendicitis is given by the appearance of appendiceal perforation: a macroscopically visible transmural defect of the appendix wall. That is understandable: appendiceal perforation has been associated with a worse outcome and a mortality rate of up to 5% (12). This definition does most probably not resemble the most reasonable differentiation criterion on a pathophysiological level – as will be demonstrated later in this article -, but at least it does not leave room for interpretation. One analysis revealed an increase in pediatric perforated appendicitis within the COVID-19 outbreak: compared with a historic baseline cohort patients a significant *relative* increase of perforations was found (37.6% vs. 22%) (8). The paper has to be interpreted against the background of the observation of a general *decrease* of uncomplicated cases, which has been made particularly in multi-center studies (1, 2, 5, 6), and leads back to an observation that was already made a few decades ago. In a very broad study including more than 56,000 patients Andersson has demonstrated that the *incidence* per 100,000 inhabitants of non-perforating appendicitis was strongly associated with age, the analyzed time period, diagnostic accuracy and the negative appendectomy rate, while the incidence of perforation appendicitis was stable without any of those associations (13). This led to the conclusion that a huge proportion of acute appendiceal inflammations resolves by itself. The observation was later qualified as “disconnect between incidence of nonperforated and perforated appendicitis” in another comprehensive study (14).

Compared to most other studies, the two latter are distinguished by two characteristics: first, they include a huge number of patients, which, second, allows the reliable analysis on the basis of absolute numbers, in contrast to relative numbers, which represent a quotient of two variables both influencing independently the result: according to Andersson, it can only be the number of uncomplicated appendicitis that leads to different results, since the absolute number of perforating appendicitis - within certain limits - remains stable. This finding is supported by a multicenter study with data collected during a 10-week interval within the COVID-19 lockdown 2020 in Germany (15). Data where compared with those from the same period in the same hospitals in 2019. The analysis of 1,915 appendectomies from 41 surgical departments revealed that the *rate* of complicated inflammation increased while the *absolute* number even slightly decreased. Morbidity and mortality were not affected. This observation of decreased incidence of uncomplicated appendicitis without an accompanying increase in complicated disease during the COVID-19 pandemic has also been made by others and led to the familiar conclusion that particularly spontaneous resolution of uncomplicated might have been responsible (1, 3, 16).

But how can this observation be explained on a pathophysiological level? Are there really two independent entities or is there a link between complicated and uncomplicated appendicitis? Might COVID-19 itself influence the course of the disease?.

In the following all these issues will be addressed based on the most conclusive information and studies. The resulting construct does not claim to reflect the absolute truth but represents a hypothesis that is able to resolve some contradictions against the background of a well-founded chain of causality.

## The physiological function of the vermiform appendix

First fundamental theories on the formation of acute appendicitis have been naturally been driven by, again, the perception of the course of the disease, which can take on dramatic proportions. And it is these dramatic trajectories that understandably have driven theories of the disease's pathophysiology. It is no coincidence that the first theories on the emergence and course of acute appendicitis and even treatment recommendations came from pathologists (17). Aware of the course of the disease, which can lead to death, a pattern of explanation emerges from the microscopic comparison of differently inflamed appendices: that of tissue inflammation that begins mildly and then necessarily ends in perforation, which causes severe disease. The recommendation: emergency appendectomy.

However, when considering the pathophysiology, it is worth taking a step back to the physiological function of the vermiform appendix. The normal function of the appendix is not easy to prove, but there are some compelling indications. The first question which has to be addressed is if the vermiform appendix fulfills any relevant function at all. A study including 57,261 children who had been undergoing appendectomy revealed that children who received appendectomy had a 2.38 times higher risk to develop sepsis (18). This finding fits into a fundamental theory of the physiological function of the appendix: the “safe house” theory. According to this theory, the appendix serves as a refuge area for the bacterial local flora of the intestine, through which the intestine can be repopulated after viral gastrointestinal infections (19, 20). Indeed, the vermiform is histopathologically distinguished from other parts of the colon by particular accumulations of lymphatic tissue. Interestingly, these accumulations consist of certain T- and B-cell populations, which is very well compatible with theories on antiviral functions (21). And even further: these cell populations can already be found in the fetal stage of development, which shows that the accumulation of these cell types is not the result of infection but is most probably constitutive and provides the prerequisite for the physiological function of appendix. All this information taken together might lead to the conclusion that the persistence of the commensal bacterial flora of the gut is widely dependent on the antiviral function of the appendix. Disturbance of this balance e.g., by surgical appendectomy might lead to an increased risk for non-physiological bacterial overgrowth and might explain the increased risk for the development of sepsis. Thus, the answer seems to be: yes, the appendix has a function.

## The inflammation of the vermiform appendix

The emergence of acute appendicitis has been attributed to most different reasons like obstruction – particularly by fecaliths – (22), infectious agents (23), hygienic factors (24), dietary aspects (25), ischemia (26), traumatic causes (27), genetic factors (28) and allergy (29). However, the pathophysiology of acute appendicitis can most probably be explained against the background of the physiological function of this part of the bowel: a constitutively antiviral function. Indeed, there is evidence pointing at viral infections as possible cause for the development of symptomatic appendicitis (23, 30, 31).

This evidence leads back to the epidemiological observation of inflammatory entities presenting with independent courses with different consequences for affected patients (13, 14). The challenge is to provide pathophysiological explanatory patterns for these observations. In an attempt to provide pathophysiological footing for their own epidemiological study results, the group around Roland Andersson performed immunological research in patients with acute appendicitis. The central finding of a comprehensive cytokine-based investigation was that patients with histopathologically gangrenous appendicitis where characterized by a substantial increase of highly inflammatory markers which are particularly associated with Th1 and Th17 cells (32, 33). IL-17 leads to rapid recruitment of neutrophils to sites of infection by promoting epithelial, endothelial, and stromal cells, each of which produce activating cytokines and chemokines. The relevance of the IL-17 immune response in patients is also reflected in other observations in patients with appendiceal inflammation (34). It has been demonstrated that IL-17 is particularly rapidly induced in infection with Escherichia coli, which represents the most commonly detected bacterial finding in acute complicated appendicitis (31). Appendectomy at a young age protects against inflammatory bowel disease, which has been attributed to suppression of the IL-23/Th17 pathway (35). Thus, some clinically relevant connections have been previously suggested.

**Figure 1:**
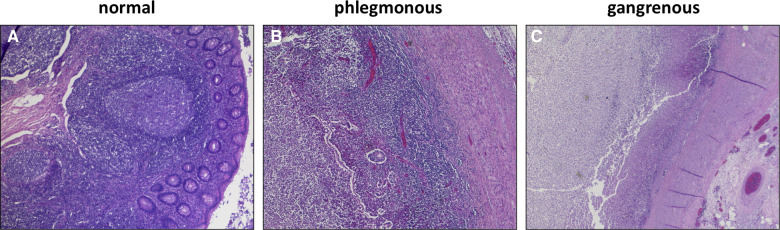
Histopathological features of the vermiform appendix (40): (**A**) Normal lymphofollicular structures of uninflamed appendix. (**B**) transmural granulocyte infiltration and oedema in phlegmonous appendicitis. (**C**) transmural necrosis and fibrinous-purulent inflammation in gangrenous appendicitis.

The crucial point in the studies which had been performed by Andersson and Rubér was that the relevant discrimination criterion was not perforation but transmural necrosis within gangrenous appendicitis with the risk for bacterial transmigration and progression to perforation (32, 33). Indeed, gangrenous appendicitis is clinically associated with significant complication rates as abscess formation, wound infection and bowel dysmotility compared with non-necrotic phlegmonous appendicitis (26, 36, 37). The mortality rate is 6 times as high (12). Analysis of differential blood counts in patients with gangrenous appendicitis showed a time stable increase of neutrophils, which are highly associated with Th17 and Interleukin-(IL-)17 dependent pathways (38). Furthermore, inflammatory course in patients with gangrenous appendicitis was characterized by a steady increase and then stabilization of c-reactive protein – in contrast to phlegmonous appendicitis with a moderate increase and then spontaneous resolution (38, 39). The histopathological features of phlegmonous and gangrenous appendicitis are demonstrated in Figure 1.

Research has so far made no substantial progress with regard to the pathophysiological explanation of acute appendicitis. Next to the disagreement regarding the relevant differentiation criteria, the investigation methods were previously defined by assumptions and correspondingly limited methodological approaches. Particularly, the RNA based genome-wide gene expression analysis enables a comprehensive consideration of basic biological processes with good temporal resolution by quantifying almost all transcriptional processes of protein biosynthesis (41). Hopeful diagnostic approaches have emerged with the use of the method, e.g., for the prediction of perioperative sepsis (42).

The method has also given interesting insights into the differential pathophysiology of histopathologically phlegmonous and gangrenous appendicitis in children. In a recent study genome-wide gene expression analysis was followed by specific immunological pathway analysis (40). The difference to previous gene expression analyses in patients with acute appendicitis was the differential investigation of necrotizing gangrenous and non-necrotizing phlegmonous appendicitis, which was assumed to be the essential difference (43, 44). Gene expression in patients with gangrenous appendicitis was particularly characterized by increase of gene expression associated with neutrophil and monocyte function. The role of the previously relevantly shown Th17-associated mechanisms in gangrenous appendicitis was particularly demarcated by the highly differential regulation of cytokines and transcription factors of the IL-23/IL-17 pathway: IL-17A, IL-23A, IL-23R, IL-27 and SOCS3 (40, 45).

**Figure 2:**
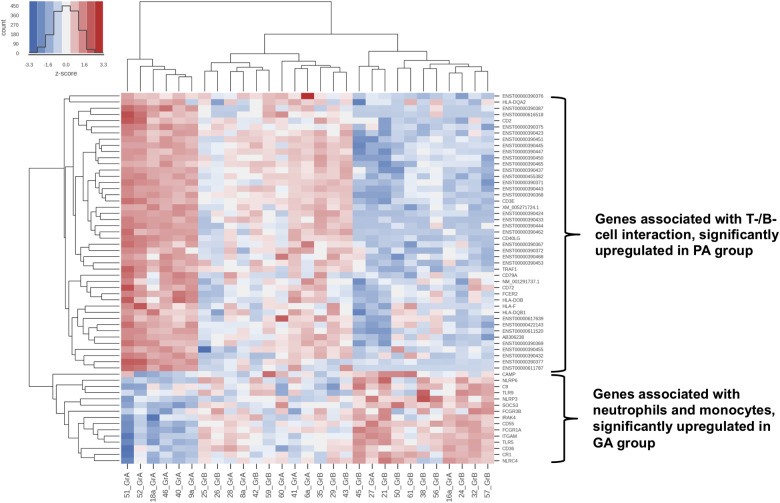
Heat map: cluster formation of samples with similar immunological patterns within genome wide gene expression analysis with RNA-microarray (40). Differentially expressed pathways according to the Generally Applicable Gene-set Enrichment (GAGE) method by Luo et al. using the “gage” package in R (48).: Toll and lmg signaling pathway, Antigen processing and presentation, NOD-like receptor signaling pathway, Hematopoietic cell lineage, Natural killer cell-mediated cytotoxicity, TNF signaling pathway and Intestinal immune network for IgA production, Complement and coagulation pathways, RIG-I-like receptor signaling pathway, Cytosolic DNA-sensing pathway, C-type lectin receptor signaling pathway, T-cell receptor signaling pathway, Th1 and Th2 cell differentiation, Th17 cell differentiation, IL-17 signaling pathway, Fc epsilon RI signaling pathway, Fc gamma R-mediated phagocytosis and Chemokine signaling pathway. (**A**) samples from patients with phlegmonous appendicitis (PA); (**B**) samples from patients with gangrenous appendicitis (GA); red colour: signal above mean signal, blue colour: below mean signal.

**Figure 3:**
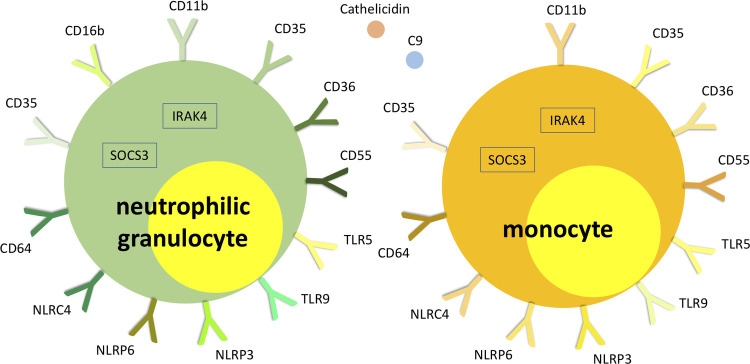
Visualization of significantly overexpressed genes related to neutrophilic granulocyte and monocyte surface proteins and transcription factors in patients with gangrenous appendicitis (40).

**Figure 4:**
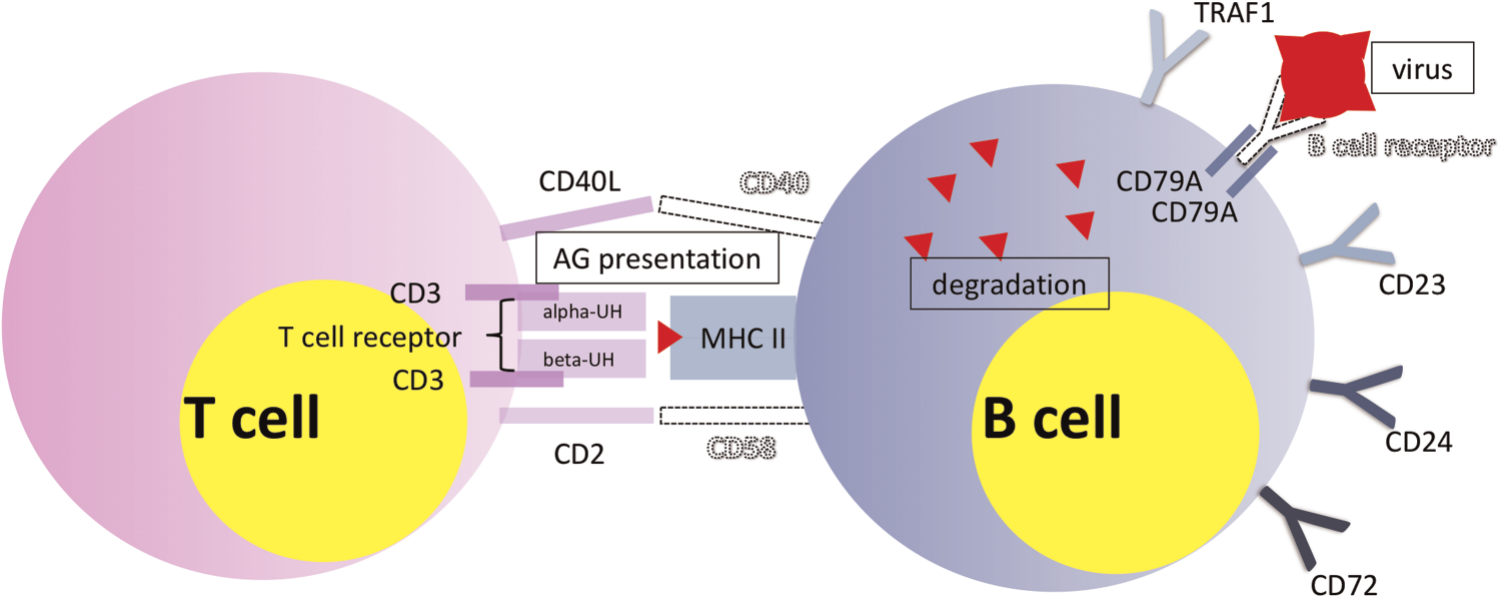
Visualization of significantly overexpressed genes associated with B and T cells, demonstrating particularly their interaction (40). Objects shown dashed and virus symbol: added for demonstration purposes.

Phlegmonous appendicitis was accompanied by an expression pattern suggesting a specific immunological synapse, which is essential for induction of an antiviral humoral response: the overexpression of mRNA of proteins of the T cell-CD3 complex and of co-stimulatory CD40l and CD2 (40). CD40l has independently been shown to be a predictor of acute appendicitis (46). The expression patterns of T cell receptor subunits are of particular importance. 33 T cell receptor subunits (alpha and beta subtypes) are *exclusively* significantly overexpressed in patients with phlegmonous appendicitis, representing 11% of the 100 top differentially expressed genes alone (40). Next to the observation of the virus associated immunological synapse this finding supports the theory of virus induced phlegmonous appendicitis, as the modulation of T cell receptor signaling and expression is a specific viral function (47). Figure 2 demonstrates the significantly overexpressed genes related to surface proteins and transcription factors associated with neutrophilic granulocytes and monocytes in patients with gangrenous appendicitis and show significantly overexpressed genes associated with B and T cells in patients with phlegmonous appendicitis after immunological pathway analysis as heat map (40). Figures 3, 4 show the corresponding proteins in their physiological context.

It has been proposed to establish gene expression based signatures for diagnostic purposes based on the smallest possible set of genes with the best possible diagnostic or predictive performance (41). By using modern methods of artificial intelligence and machine learning it was possible to define a gene expression based biomarker signature consisting of 4 genes best performing for differentiation of phlegmonous and gangrenous appendicitis in children within an experimental approach (49).

Thus, substantial evidence qualifies necrotic gangrenous appendicitis as a bacterial and non-necrotic phlegmonous appendicitis as viral disease – the latter particularly against the background of its probable originate function as antiviral unit.

The difficult question remains whether there is an association between uncomplicated and complicated appendicitis. The above suggests two completely independent entities. There is the frequent perception that obstruction of the appendix lumen might be a leading course of acute appendicitis (26). In fact obstruction appears in only a minority of patients with acute appendicitis patients and especially fecaliths are regular findings in normal appendices without inflammation (26, 50). On the other hand, particularly perforated appendicitis has been associated with the presence of fecaliths (51), while in a recent analysis on the discriminatory capacity of ultrasound the presence of a fecalith showed a low specificity for presence of gangrenous appendicitis (52). However, as one study encounters fecaliths as incidental findings in one third of autopsies (53), it is theoretically conceivable that the constant incidence of the presence of fecaliths justifies a constant incidence of complicated appendicitis on the basis of a significantly larger number of phlegmonous appendicitis.

## Are there clinical implications for treatment of acute appendicitis in children within the COVID-19 pandemic?

Although a pathophysiological correlation of acute appendicitis with Covid-19 associated pediatric pathologies like Kawasaki Disease and Pediatric Multisystem Inflammatory Syndrome has been discussed in single case reports (54–56), a causal relationship between those two is not likely – especially due to the fact that conditions of complicated appendicitis remained most probably stable and the incidence of uncomplicated inflammation decreased (1–5). Possibly there might be even a negative correlation, as there was a particular decrease of viral infections during the pandemic, which might have influenced the incidence of acute appendicitis in general and specifically of phlegmonous inflammation (57). This hypothesis is supported by the decrease of hospital admissions of children with (other) virally triggered conditions like asthma (58).

A central clinical aspect which had been addressed frequently during the pandemic is a possible delay of diagnosis and related deterioration of the affected patientś clinical conditions (9, 10). Especially children have been reported to be affected (8, 11). Again, these results can be looked at against the background of pre-existing studies on the influence of a delay of diagnosis and treatment of acute appendicitis. Existing evidence contradicts the thesis that the timing of intervention has a relevant influence on the outcome or length of stay irrespective of age group, at least if the delay does not exceed 24 h (59–61). In accordance with these results several studies could not confirm the observation of an average late presentation of patients with acute appendicitis within the COVID-19 pandemic. Even if delayed treatment was found, the treatment results were not affected compared to pre-pandemic times (2, 4, 5, 7, 62).

In summary, especially against the background of existing evidence it can be said that the COVID-19 pandemic has most likely no causal relationships with acute appendicitis in children and adults that go beyond the described. However, current changes in epidemiological conditions within the pandemic allow for a discussion about basic conditions and prerequisites of the disease. It remains to be seen whether the insights gained during the pandemic will actually have an impact on future therapeutic modalities, especially with regard to conservative treatment strategies (16, 63).
